# An *in vitro* simulation method for the tribological assessment of complete natural hip joints

**DOI:** 10.1371/journal.pone.0184226

**Published:** 2017-09-08

**Authors:** Dawn Groves, John Fisher, Sophie Williams

**Affiliations:** Institute of Medical and Biological Engineering, School of Mechanical Engineering, University of Leeds, Leeds, United Kingdom; Rush University Medical Center, UNITED STATES

## Abstract

The use of hip joint simulators to evaluate the tribological performance of total hip replacements is widely reported in the literature, however, *in vitro* simulation studies investigating the tribology of the natural hip joint are limited with heterogeneous methodologies reported. An *in vitro* simulation system for the complete natural hip joint, enabling the acetabulum and femoral head to be positioned with different orientations whilst maintaining the correct joint centre of rotation, was successfully developed for this study. The efficacy of the simulation system was assessed by testing complete, matched natural porcine hip joints and porcine hip hemiarthroplasty joints in a pendulum friction simulator. The results showed evidence of biphasic lubrication, with a non-linear increase in friction being observed in both groups. Lower overall mean friction factor values in the complete natural joint group that increased at a lower rate over time, suggest that the exudation of fluid and transition to solid phase lubrication occurred more slowly in the complete natural hip joint compared to the hip hemiarthroplasty joint. It is envisaged that this methodology will be used to investigate morphological risk factors for developing hip osteoarthritis, as well as the effectiveness of early interventional treatments for degenerative hip disease.

## Introduction

Osteoarthritis (OA) causes pathological degenerative changes that affect the whole joint [1] leading to pain and loss of function [2, 3]. Primary total hip replacement (THR) is often used to alleviate the pain and disability caused by advanced OA of the hip, however, many younger patients require revision surgery after 15–20 years, and outcomes following this procedure are not always as favourable in terms of patient satisfaction and function [2, 4]. It is hypothesised that tribological studies of complete natural hip joints using *in vitro* simulations would be of benefit for investigating relationships between hip geometry and degenerative joint disease such as OA, as well as investigating the efficacy of early interventional treatment, which may delay the onset of OA [5–8].

*In vitro* simulation studies investigating the tribology and function of THR prostheses are widely reported in the literature, however, studies exploring the friction and wear between two contacting natural cartilage surfaces have been focussed largely on reciprocating motion friction studies using cylindrical osteochondral plugs [9–11]. Despite the natural hip joint being relatively congruent, contact between the two articulating surfaces changes under different weight-bearing conditions and the femoral head is slightly spheroidal in shape compared to the more spherical acetabulum [12]. This makes biomechanical analysis of the natural hip joint more complex compared to the artificial hip joint and therefore to date, research investigating complete natural hip joint tribology *in vitro* has been limited and heterogeneous in nature [13–18]. Tribology of the hip joint following hemiarthroplasty, where only the femoral head is replaced with a prosthesis, has also been investigated experimentally using both *in silico* and *in vitro* methods [19–21], albeit to a lesser degree than THR tribology.

The main aim of this study was to develop a complete *in vitro* simulation model, initially using natural porcine hip joints and a pendulum friction simulator, in order to investigate the tribology of the complete natural hip joint. The method was developed to facilitate hips with different morphologies, and to be easily modifiable for use with human tissue and different simulation systems, e.g. a physiological hip joint simulator. The methodology was assessed by conducting *in vitro* simulations on a group of complete, anatomically matched porcine hip joints and a group of porcine hip hemiarthroplasty joints, and with the intent of testing hip joints positioned with different acetabula and femoral orientations in future studies. The data associated with this paper is available from the University of Leeds Data Repository [22].

## Materials and methods

### Pendulum friction simulator

**Fig 1:**
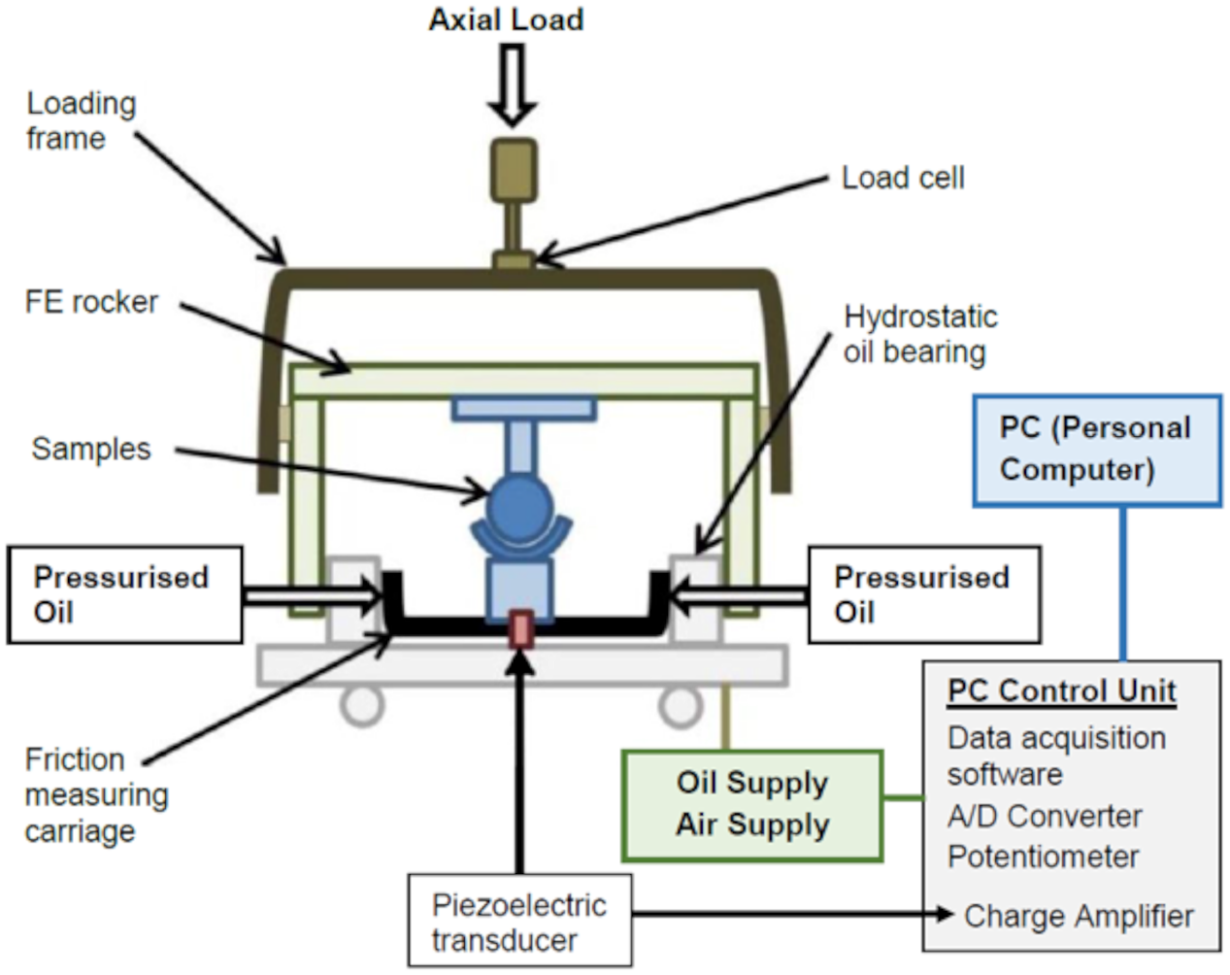
Pendulum friction simulator. Schematic diagram of the pendulum friction simulator. FE: flexion-extension; A/D: analogue to digital converter.

A ProSim pendulum friction simulator (Simulation Solutions Ltd., Stockport, UK), which is a pneumatically loaded single station simulator, was used for the *in vitro* simulations in this study (Fig 1). Hip joints were inverted with respect to anatomical position in the simulator, which applied an axial load through the femoral head and applied motion via a flexion-extension (FE) rocker. A piezoelectric force transducer attached to the front of a self-aligning friction measuring carriage, and therefore aligned with the FE axis, measured any forces transferred between the bearing surfaces as the FE rocker moved back and forth. The carriage itself was mounted on a pressurised hydrostatic oil bearing and was designed so that any torque created by sample misalignment and incidental movement of the carriage in a medial-lateral direction, i.e. not due to friction between the two surfaces arising from FE motion, would be negligible. This was important for testing biological tissue that may not be of uniform and/or symmetrical geometry, as this could give rise to additional torque from concomitant movement of the carriage. The magnitude of FE frictional torque created by the samples being tested was determined by converting the force data from the piezoelectric transducer into a voltage signal using a charge amplifier. The transducer was able to measure frictional torque to a minimum value of 0.5 Nm (1% of the maximum range of the transducer, which was 50 Nm), with friction factors measurable in the range of 0.01–0.5 [23].

### Natural tissue fixture design

Fixtures were developed to facilitate the orientation and positioning of natural acetabula with varying angles of version and inclination, and natural femoral heads to be positioned with different combinations of angles in all three planes. The risk of experimental artefact was reduced by enabling natural hips of different sizes to be potted centrally with the centre of rotation (COR) of the head and acetabulum aligned with the pendulum friction simulator. The position of the samples in the simulator was checked using an alignment rod (supplied by the manufacturer), which was designed to pass through holes in the FE rocker and friction measuring carriage, arranged in series corresponding to the COR of the simulator.

#### Acetabulum

**Fig 2:**
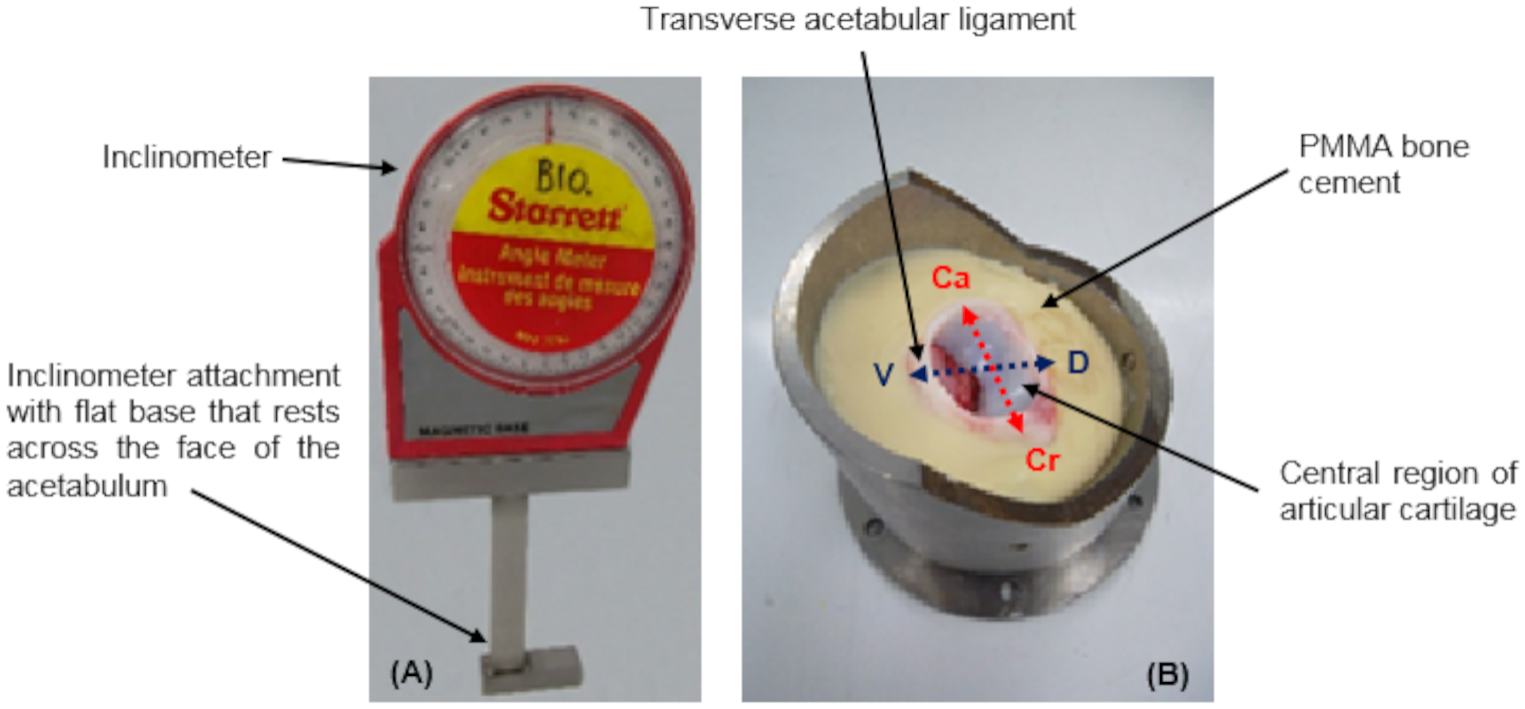
Porcine acetabulum potted using an inclinometer. (A) Inclinometer with attachment used to orientate and position the acetabulum and (B) potted porcine acetabulum showing the position of the specimen in the test pot. The dorsal—ventral and cranial—caudal directions correspond to the inclination and version angles respectively. PMMA: polymethyl methacrylate; D: dorsal; V: ventral; Cr: cranial; Ca: caudal.

A test pot allowing sufficient access and control over the acetabulum during potting and removal of the specimen, whilst also reducing the risk of impingement between the components during testing, was designed and manufactured from stainless steel. A potting methodology using an inclinometer was developed, which provided a consistent and repeatable way of orientating the acetabulum whilst controlling the degree of inclination and version to be applied to the acetabulum (Fig 2). This method provides two independent variables that can be used in future *in vitro* simulations to replicate different *in vivo* morphologies, for example a retroverted acetabulum.

#### Natural femoral head potting jig

**Fig 3:**
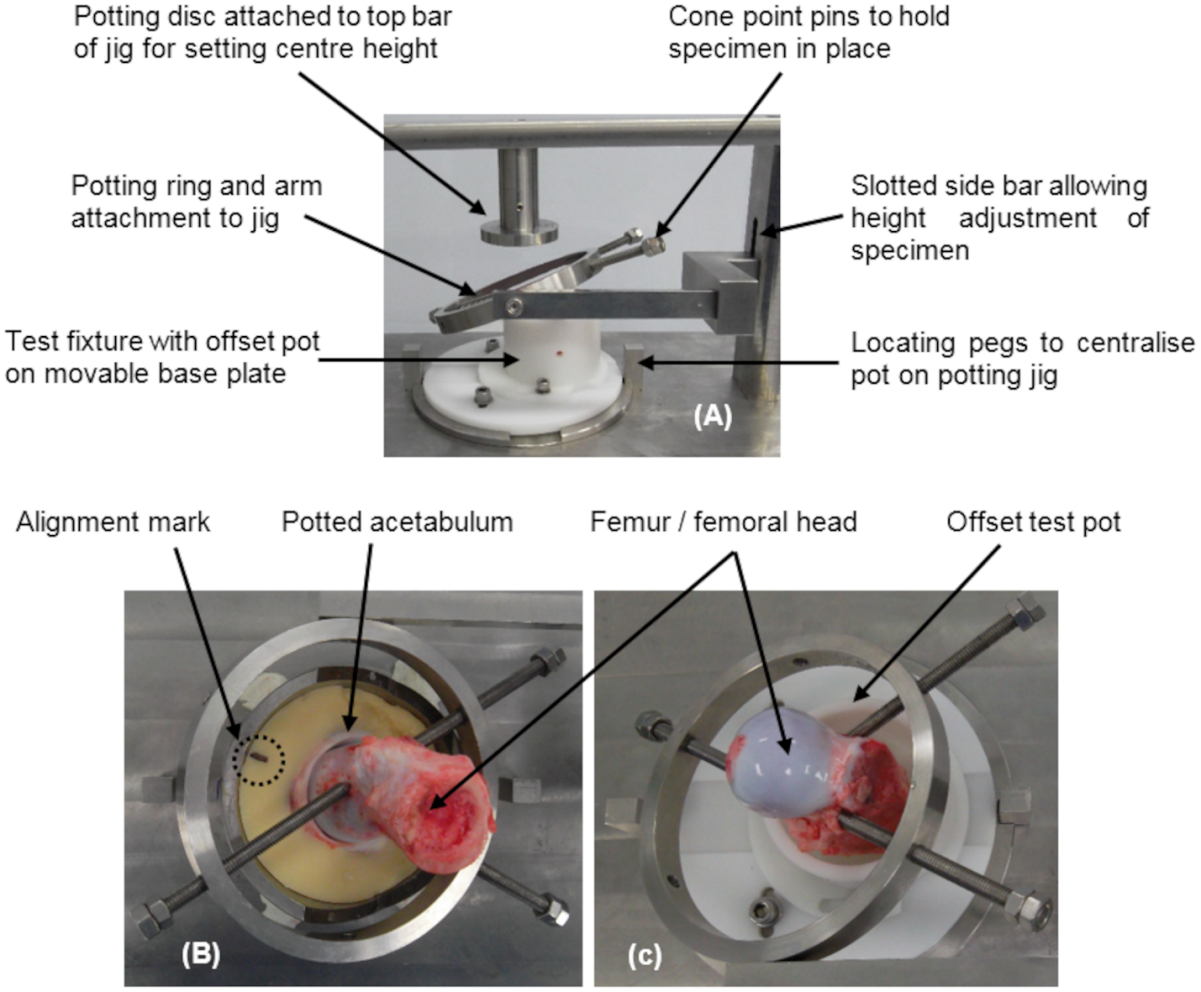
Femoral head test pot and potting fixture. Femoral head test pot and potting fixture (A) with (B) the porcine femoral head orientated and positioned using the acetabulum and (C) the femur positioned in the test pot before cementing, held in position using the potting ring and cone point pins.

The potting jig was a modular design with interchangeable fixtures enabling femoral heads of different diameters to be correctly positioned. Potting discs of varying depths, designed to be used with femoral heads of different radii, were manufactured and the selected disc was vertically aligned with the centre of the test fixture base, once attached to the top bar of the potting jig (Fig 3A). The jig was designed so that once fully assembled, positioning the superior surface of the natural femoral head against the underside of the disc, aligned the centre of the head with the centre height of the FE rocker, to which the femoral sample was attached during the test. The potting jig enabled movement between the ring and the arm (inclination), and between the assembly attachment and a slotted base post (version), thus enabling the position and orientation of the sample to be controlled.

#### Natural femoral head test fixture

The test fixture consisted of a Delrin^®^ test pot, offset to accommodate the anatomical offset of the femur whilst maintaining vertical alignment of the head with the direction of axial loading from the simulator. Samples were held in place during positioning and potting using a potting ring and cone point pins (Fig 3B and 3C). The test pot was secured to a Delrin^®^ top plate and stainless steel base plate, designed to be moved in two orthogonal directions so that different femoral shaft sizes/shapes and positions could be accommodated.

#### Natural tissue sample preparation

Hip joints from porcine right hind legs were harvested from 25 week old donor pigs, with an average weight of 80 kg, 24–48 hours following slaughter at a local abattoir. Hip joints received from the abattoir had only minimal and varying amounts of pelvic bone attached, and therefore, the orientation of the joint in this study was defined by the relationship to the reference frame of the simulator. The horizontal simulator base formed the transverse plane, and the anteroposterior axis and FE motion occurred in the sagittal plane. Cartilage surfaces were kept hydrated with phosphate buffered saline throughout the harvesting and potting procedures. The diameter of the hip joints were measured using the harvested femoral head and a set of circular gauges. As the heads were slightly aspherical, size selection was based on there being no interference of the femoral head from the gauge in the cranial—caudal direction, which corresponded to the FE direction of motion. This diametric measurement was used to select the correct sized potting disc for the complete natural joint studies, and for selecting a size-matched cobalt chrome (CoCr) metal head for the hemiarthroplasty studies.

Simulations for the complete natural hip and hip hemiarthroplasty studies were conducted with the acetabula positioned with the same orientation so that between-group comparisons of friction factor could be made.

Harvested tissue was potted using a three-stage process, and all tissue samples were secured in their respective test pots using polymethyl methacrylate (PMMA) bone cement.

1. Acetabula were positioned and potted with the transverse acetabular ligament uppermost and central region of the articular cartilage inferiorly. Samples were centred and aligned with the COR of the simulator using a potting jig previously developed by Lizhang [24], that had been modified to accommodate the manufactured acetabulum test pot [25]. In brief, this custom made rig consisted of a base plate, on which the acetabulum pot was centred, and a vertical track with moveable collar. The size-matched CoCr head was attached to a rod, which was clamped via an arm onto the vertical track, and lowered onto a setting block before fixing the collar in place. A range of setting blocks to be used with heads of different radii were available and designed so that once the arm was resting on the collar, the centre of the CoCr head matched the centre height of the friction measuring carriage, which was where the acetabulum was seated during the test. The setting block was replaced with the acetabulum pot and the acetabulum, which was placed into PMMA cement whilst it was in a workable state, was pushed gently down into the cement using the femoral head until the arm rested on the collar. This aligned the centre of the acetabulum with the simulator, and the inclinometer was used to concurrently position all samples with neutral version and an inclination angle equivalent to 45° (Fig 2B). Acetabular version and inclination were defined as the angle between the acetabular rim plane and the sagittal and transverse planes of the simulator respectively. This acetabula orientation replicated the default set-up used in a study of porcine hip hemiarthroplasty joints by Lizhang et al. [20], thus enabling the mean friction factor values in this group to be compared with those from this previously published tribological study.
2. Porcine femurs were positioned in the potting ring so that the articulating surfaces of the femoral head and acetabulum were congruous, and with the head in anatomical alignment with the potted acetabulum. This was achieved using an alignment mark placed opposite the midpoint of the transverse acetabular ligament and bony reference point on the femoral neck (Fig 3B). The bony reference point was a linear elevation, running inferiorly between the equator of the femoral head and the distal end of the intertrochanteric ridge, just above the lesser trochanter. These two landmarks were identified as being repeatable anatomical reference points that could be used to align the joint during exploratory work, which was conducted prior to the development of this methodology.
3. The potted acetabulum was replaced with the femoral head test pot fixture and the femur was inverted, whist maintaining its orientation from Stage 2 in the potting ring. The femoral shaft was positioned centrally in the test pot of the head test fixture (Fig 3C), and the COR was obtained by raising the head up to the correct diametral sized potting disc (Fig 3A). The shaft was secured by pouring PMMA cement into the test pot, which was allowed to fully cure before removing the potting ring, ensuring that the sample didn’t move once positioned.

### Hemiarthroplasty model

Hemiarthroplasty tests were conducted using natural acetabula and size-matched CoCr femoral heads (DePuy Synthes, Leeds, UK). Acetabula were potted using the method described above, and the CoCr heads were mounted and tested using an existing fixture. This consisted of a moveable vertical spigot that could be adjusted to set to the correct COR using slip gauges and a Vernier height gauge.

### Measurement of friction

**Fig 4:**
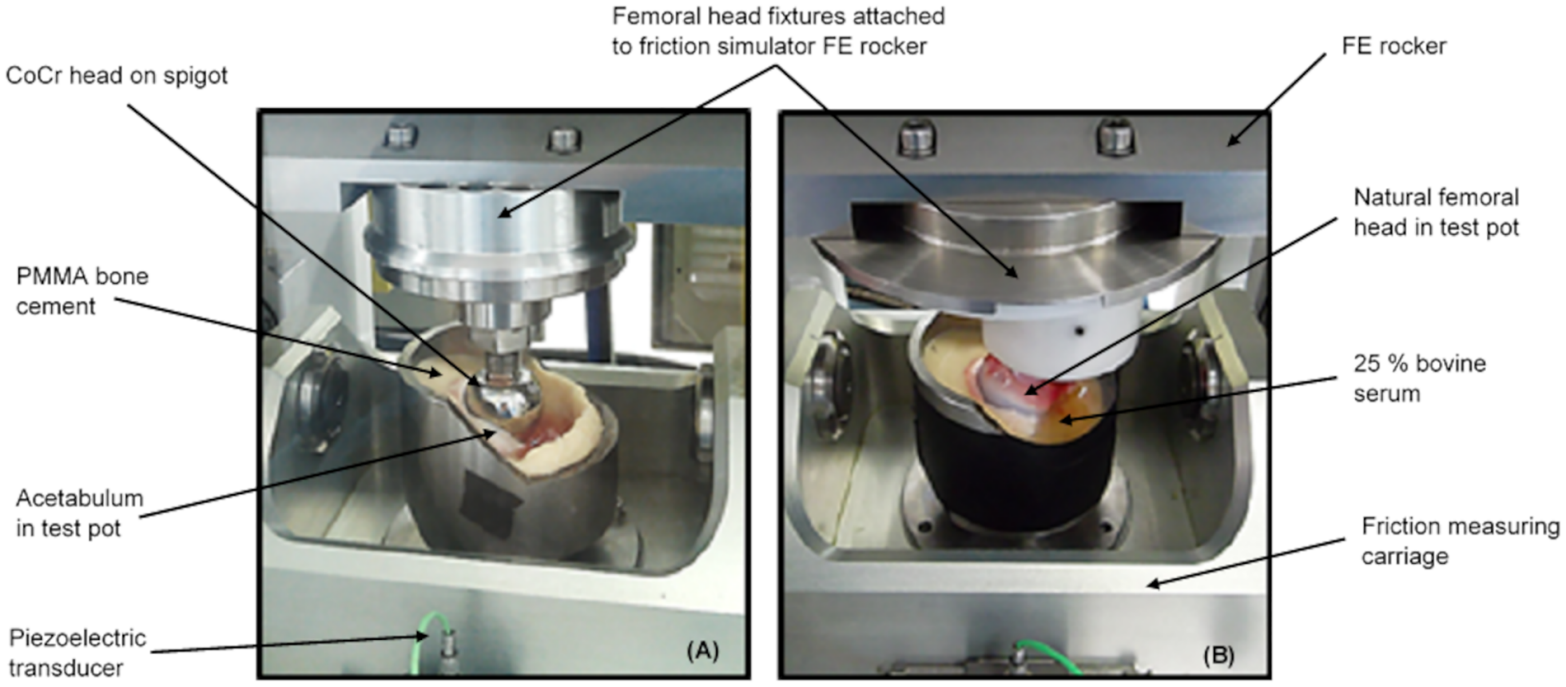
Hemiarthroplasty and natural joint samples. Pendulum friction simulator with (A) porcine hemiarthroplasty and (B) complete porcine hip joints *in situ* with 25% bovine serum lubricant before testing.

*In vitro* simulations were conducted on porcine hemiarthroplasty specimens (n = 5) and complete, matched natural porcine hip joints (n = 5). Samples were fixed into the simulator with the acetabula pot seated in the friction measuring carriage and the head attached to the FE rocker (Fig 4). Once in the simulator, the position of the samples was checked by passing the alignment rod through the holes in the FE rocker and friction measuring carriage. Tests were only conducted if the rod passed through both alignment holes. Friction was determined via the piezoelectric transducer (Fig 1), which measured the frictional torque generated between the contacting surfaces of the femoral head and acetabulum. The lubricant for all tests was a 25% volume-to-volume concentration of newborn calf serum, diluted with deionised water, which had a protein content similar to human synovial fluid [26].

**Fig 5:**
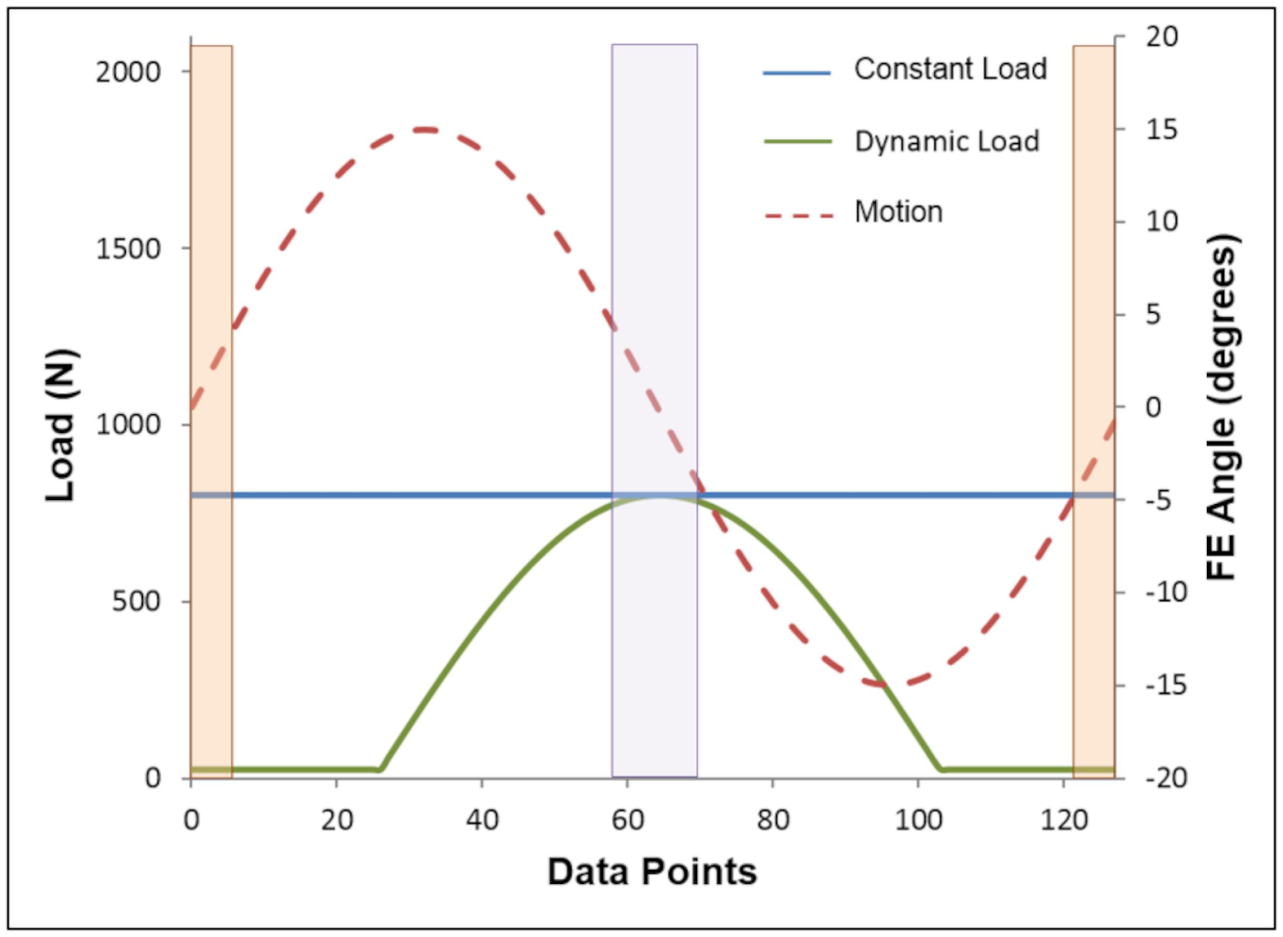
Simulator motion and loading profiles. Motion and dynamic (25 N to 800 N) and constant (800 N) loading profiles for one cycle. The orange shaded area shows where data is gathered at the mid-flexion and mid-extension points during the constant load pre and post-tests, and the purple shaded area shows the peak load (i.e. 800 N) high velocity phase where the dynamic profile test data is collected from. FE: flexion-extension.

Loading and motion conditions previously reported for testing porcine hemiarthroplasty samples were used [20], where simulations were run at a frequency of 1 Hertz for 7,200 cycles (i.e. 2 hours). A single sinusoidal dynamic load ranging from 25 N (swing phase) to 800 N (stance phase peak load) was applied through the femoral head, whilst simultaneously applying ± 15° FE motion (Fig 5). The input profile simulated non to full weight-bearing through one hind limb of a donor pig weighing on average 80 kg. The loading profile was designed to be comparable with the loads experienced through the hip joint during a quadrupedal gait cycle [27–29], and due to pigs having a smaller range of motion during normal gait than bipedal humans [29, 30], a motion of ± 15° of flexion to extension was used to reduce the risk of any bony impingement. To account for any additional frictional torque arising from misalignment due to the complex geometry of the joint, the data was normalised using a mean frictional offset value calculated from two-minute 800 N constant load tests (± 15° FE), which were conducted before (pre-test) and after (post-test) each dynamic profile study. Pre and post-test frictional torque was measured during mid-flexion and mid-extension (Fig 5), which enabled any differences in flexion and extension torque measurements arising from off-centre loading of the aspherical samples to be accounted for [20].

### Data analysis

Friction factor (*f*) was calculated using Eq (1) from the true torque magnitude (T_t_) detected by the piezoelectric transducer, where r is the radius of the bearing surfaces (meters) and *W*_p_ is the peak load (Newtons):

$$f=\frac{T_{t}}{r\times W_{p}}$$ (1)

**Fig 6:**
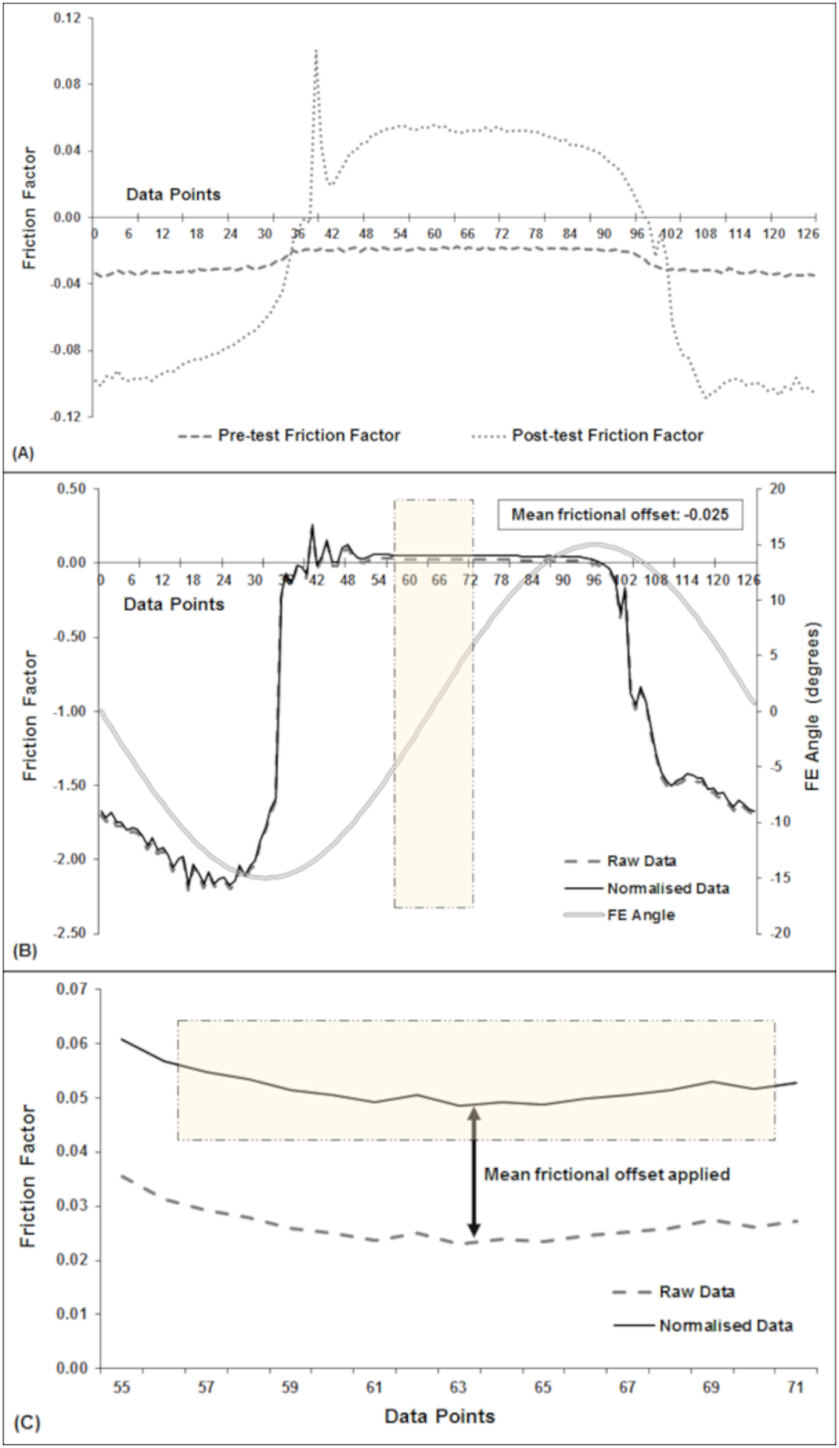
Data plots taken from a hemiarthroplasty test showing normalisation of the friction factor using constant load test data during the post-processing stage. (A) Data plots of the friction factor measured during one cycle of the 2-minute 800 N constant load tests conducted before (pre-test) and after (post-test) the dynamic profile study, which was used to calculate the mean frictional offset value (*f*_o_). The mean frictional offset was -0.025 in this example, which was calculated using Eq 2. (B) Raw data plot of the friction factor measured during a single cycle of a dynamic profile study plotted against the same data set with the mean frictional offset value applied (i.e. normalised) using Eq 3, and (C) the section of the graph where the normalised data (*f*_n_) for the reported values is taken post-processing. The pale yellow shaded areas highlight the data collection area. FE: flexion-extension.

The mean friction factor for each one-second cycle of the dynamic load tests, was calculated from the data collected during the peak load (i.e. 800 N) high velocity phase, and friction factor for the constant load tests (example raw data plots shown in Fig 6A) was calculated using data taken from where the head was vertically loading the cup, (i.e. 0° FE), corresponding with the high velocity phase of the cycles, as shown in Fig 5. Mean frictional offset (*f*_o_) was calculated using data from the constant load tests conducted before (*f*_b_) and after (*f*_a_) each dynamic profile study Eq (2):

$$f_{o}=\frac{f_{b}+f_{a}}{2}$$ (2)

Friction data was normalised (*f*_n_) by subtracting the frictional offset (*f*_o_) from the mean dynamic friction factor (*f*_d_) of each logged cycle Eq (3) to give a friction value for the test:

$$f_{n}=f_{d}-f_{o}$$ (3)

Example raw data plots measured during a single simulator cycle and the corresponding normalised dynamic profile test plot, adjusted for mean frictional offset following post-processing, are shown in Fig 6.

Mean friction factor values and 95% confidence limits (CL) throughout the two-hour tests was calculated, and a two-way analysis of variance (ANOVA) was carried out on friction factor by bearing couple (i.e. hemiarthroplasty and complete joint), and time (i.e. cycle number) using SPSS predictive analytics software (version 19, IBM, New York, US), where p < 0.05. Time was discretised into three levels and analysed at the one-minute (start; 60 cycles), two-hour (end; 7200 cycles) and twenty-minute (1200 cycles) time points. The latter was an arbitrary time point enabling data from the initial stages to be compared with data from the start and the end of the tests.

In addition to the quantitative analysis of friction, a qualitative macroscopic evaluation of the specimens’ articulating cartilage surfaces was conducted, both before and after the simulation, in order to identify any visible damage or changes in surface appearance following testing.

## Results

**Fig 7:**
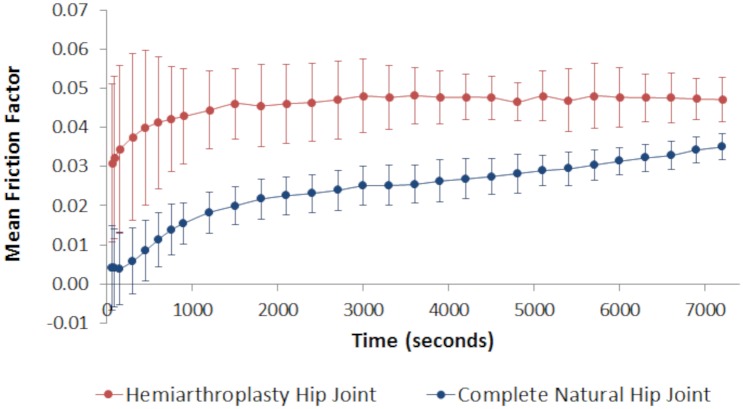
Mean friction factor for hemiarthroplasty and complete natural joint groups. Mean friction factor ± 95% confidence limits for the complete porcine hip joints (n = 5) and porcine hip hemiarthroplasties (n = 5) tested in the pendulum friction simulator for two-hours.

Complete natural porcine hip joints (n = 5) and porcine hip hemiarthroplasties (n = 5) with a diametral range of 35–37 mm were successfully positioned with the required anatomical orientation and joint centre height, enabling *in vitro* tribological testing to be conducted in the pendulum friction simulator. During the two-hour test period, an initial rapid increase followed by gradual rise in friction factor was observed for all samples in both groups (Fig 7). Mean friction factor in the hemiarthroplasty group was 0.031 ± 0.020 at the start of the test and then increased, plateauing at 0.047 ± 0.006 after ∼1,500 cycles (i.e. ∼25 minutes). Friction factor for the complete porcine hip joint group increased from an initial mean value of 0.004 ± 0.011 to a mean value of 0.022 ± 0.003 during the same time period, however, the friction factor did not plateau and continued to gradually increase with a value of 0.035 ± 0.003 being recorded after two hours. The effects of both bearing couple and time had a statistically significant effect on the friction factor (p < 0.001), however, the interaction between these two variables was not significant (p = 0.109). Throughout the two-hour *in vitro* simulations, the mean friction factor was lower in the complete natural hip group when compared to the hip hemiarthroplasty group, and this was significantly different at the 60, 1200 and 7200 second time points (ANOVA; p<0.001). A Bonferroni post hoc analysis showed that friction factor at the 60 second time point was significantly different to the friction factor recorded at both the 1200 second and 7200 second time points (both p<0.05), however, there was no significant difference in friction factor when comparing the 1200 and 7200 second time points (p = 0.139).

Changes in the appearance of the lunate surface and small areas of chondral damage were observed during the macroscopic evaluation of the tested acetabula. This was largely discoloration and small superficial scratches, which were evenly dispersed across the lunate surface of complete natural joint specimens, but located more centrally on the hemiarthroplasty specimens. There was also evidence of slightly deeper chondral lesions on the tested hemiarthroplasty specimens, with small fissures being observed on two of the acetabula. Some slight discoloration was observed superiorly on some of the tested natural femoral heads.

## Discussion

Osteoarthritis is a common form of degenerative joint disease, and it is generally accepted that the direct and indirect healthcare costs from treating hip OA using THR are expected to rise due to an aging population [31, 32]. Investigative studies exploring associations between hip joint morphology and risk factors for developing OA, and research studying the effectiveness of early interventional techniques for the treatment of hip OA, are therefore important areas of study for addressing both the increasing socioeconomic burden of the disease and for improving patient outcomes.

Historical pendulum studies of the natural hip joint have been reported in the literature by Unsworth et al. [18], O'Kelly et al. [16], and Roberts et al. [33], however, the experimental methodology does not appear to facilitate the testing of specimens using different orientations or varying geometric parameters, which is necessary in order to replicate different hip joint morphologies. Studies of complete natural hip joint tribology, conducted using servo hydraulic testing systems, have been reported by Ferguson et al. [13], who performed creep-consolidation tests using constant and cyclic loads before and after labral resection, and by Song et al. [17], who measured resistance to rotation. This was performed by applying rotational displacement with an axial compressive load, also before and after labrectomy, over ten 13-second cycles. Cadaveric human hip joints were investigated in both of these studies; however, not all of the test parameters were physiological.

In this study, an *in vitro* simulation system for the complete natural hip joint with potting fixtures enabling the orientation of both the femur and acetabulum to be controlled to simulate different joint morphologies, and with the joint COR aligned with that of the simulator, was successfully developed. The new fixtures and methodology were assessed by conducting *in vitro* simulations on porcine hip joints in a pendulum friction simulator so that mean friction factor values could be analysed and appraised. The potting methodology that was developed enabled both complete natural hip, and hip hemiarthroplasty joints, to be positioned and tested in the simulator with the joint COR aligned with that of the simulator. Setting the correct COR, together with the normalisation of the data to account for any extra biomechanical torque not due to the application of the loading profile, ensured that as far as possible any experimental artefact arising from the set-up or complex hip geometry was reduced.

Friction factor measured during the hemiarthroplasty tests displayed similar trends and values to those reported by Lizhang et al. [20], in a similar study of hip hemiarthroplasty tribology where extra-large clearances were used. In the complete natural joint group, mean friction factor increased from 0.004 to 0.035, with an overall mean value for the two-hour test of 0.022. Heterogeneous methodologies and the use of cartilage from different anatomical locations make it difficult to make direct comparisons between this study and previously published *in vitro* cartilage-on-cartilage tribological studies, however, the results do fall within the range of values (0.003 to 0.08) for friction between two cartilage surfaces that have been reported in the literature [10, 11, 18, 33–35].

The non-linear time response observed in both sample groups is most likely attributed to the viscoelastic response and biphasic nature of the cartilage, where, as the fluid support decreases the load gradually transfers to the solid phase [36, 37]. Friction factor did not plateau in the complete natural hip joint group indicating that the samples had not reached equilibrium at the end of the two-hour test. This trend is consistent with data obtained by McCann et al. [35] who studied *in vitro* friction in the natural knee joint. The natural hip in this study set-up has a spatially varying and time dependent load on the femoral head cartilage compared to the spherical CoCr head of the hemiarthroplasty. This means that exudation of fluid from the cartilage will have been slower, and hence the friction factor was lower and took longer to rise compared to the hemiarthroplasty model. These findings are reinforced by the pin-on-plate work of Forster et al. [38]. Additionally, fluid trapped between the deforming asperities of two cartilage surfaces, compared to having only one cartilage surface (i.e. acetabulum) in the hemiarthroplasty group, would have slowed down fluid exudation [39]. Porcine hip geometry is slightly more aspherical than that of human hips and it is acknowledged that this could have affected the results of the hemiarthroplasty tests. The difference between two diametral measurements taken orthogonally in the anteroposterior and superoinferior directions from porcine and human femoral heads with no obvious pathology (both n = 6), was reported to be 3.53 mm ± 1.78% and 1.0 mm ± 1.2% respectively [40]. It is reasonable to assume that a similar degree of asphericity exists in the articulating surfaces of the paired acetabula. Poorer conformity between the metallic, spherical CoCr head and the native cartilage of the aspherical porcine acetabulum could, therefore, result in areas of high contact stresses that are unevenly distributed around the acetabulum. This may explain why higher mean friction values and a greater degree of acetabular chondral damage was observed in the hemiarthroplasty group when compared to the complete natural hip joint group, where the acetabulum was articulating against a natural femoral head. The poorer congruity and distribution of the load observed in the hemiarthroplasty model could lead to abrasive wear and erosion of the acetabular cartilage over time, which is consistent with clinical findings [41–43].

The main limitation of using a pendulum friction simulator for conducting the simulations was that an axial load was applied through the femur, rather than through the acetabulum of the pelvis, and only one axis of motion (flexion-extension) was applied. Consequently, the normal loading and osteokinematics that the hip joint is normally subjected to *in vivo* (e.g. flexion-extension, abduction-adduction, medial and lateral rotation) could not be reproduced *in vitro*, which may result in some abnormal stresses being applied to the joint. Additionally, although the friction factor data was normalised to account for any slight misalignment of the joint, this process did not factor in any potential damaging effects that this may have had on the articulating surfaces of the joint. These factors may explain the minor areas of damage that were observed on the lunate surface of the native acetabula after running the complete natural joint simulations for only a relatively small number of cycles (i.e. over two hours).

Bovine calf serum has been used as a lubricant extensively in tribological investigations of the hip joint, however, it has been acknowledged that the viscosity and composition varies from synovial fluid, which is much more complex and contains hyaluronic acid, different proteins, enzymes and lipids [44]. Hyaluronic acid gives synovial fluid its viscoelastic properties [45], and surface phospholipids have been shown to contribute to articular cartilage boundary lubrication [46], meaning both are important factors when considering natural joint lubrication. It was beyond the scope of this study to address this, however, it is proposed that a lubricant containing hyaluronic acid and phospholipids, which together have been reported to reduce friction in cartilage models [47], should be considered in future natural joint tribological studies. Additionally, using tissue sourced from a slaughtered animal could potentially affect the lubrication regime of the joint, particularly that of boundary lubrication. This is largely due to the absence of viable chondrocytes that produce and maintain the extracellular matrix [37]. Nonetheless, these limitations did not detract from the main aim of this study, which was to develop an *in vitro* simulation system for the natural hip joint with potting fixtures enabling the orientation of both the femur and acetabulum to be controlled in future studies.

In future work, this methodology will be adapted for use with *in vitro* simulation systems that are able to simulate a more physiological movement, and this will provide a robust system for testing complete natural animal and/or human hip joints. The continuous cyclic loading used throughout the simulation, where the cartilage is unloaded for only relatively short periods during the swing phase, is representative of continuous walking, unlike input profiles that use a stop-dwell-start protocol to facilitate periods of relaxation and re-hydration of the cartilage matrix. If longer duration tests are to be considered in the future, then this type of simulation profile could provide a more realistic *in vivo* representation of activities of daily living that could be generalised to the wider population [48, 49]. Additionally, different hip joint morphologies relating to the acetabulum (e.g. retroversion, steep inclination angle), and proximal femur (e.g. varying degrees of femoral version), will be simulated and explored by using the fixtures designed in this study to vary the orientation of the acetabulum and/or femur. The use of pelvic and femoral coordinate systems, for example as defined and recommended by the International Society of Biomechanics [50], will be important for facilitating this work in future studies. This will enable morphological risk factors for the development of hip OA to be investigated by simulating different hip geometries and pathologies, as well as pre-clinical testing of early interventional treatments for hip OA.
